# Hindlimb unloading causes regional loading-dependent changes in osteocyte inflammatory cytokines that are modulated by exogenous irisin treatment

**DOI:** 10.1038/s41526-020-00118-4

**Published:** 2020-10-07

**Authors:** Corinne E. Metzger, S. Anand Narayanan, Peter H. Phan, Susan A. Bloomfield

**Affiliations:** 1 Department of Health and Kinesiology, Texas A&M University, College Station, TX USA; 2 Department of Medical Physiology, Texas A&M Health Science Center, Temple, TX USA

**Keywords:** Physiology, Cytokines

## Abstract

Disuse-induced bone loss is characterized by alterations in bone turnover. Accruing evidence suggests that osteocytes respond to inflammation and express and/or release pro-inflammatory cytokines; however, it remains largely unknown whether osteocyte inflammatory proteins are influenced by disuse. The goals of this project were (1) to assess osteocyte pro-inflammatory cytokines in the unloaded hindlimb and loaded forelimb of hindlimb unloaded rats, (2) to examine the impact of exogenous irisin during hindlimb unloading (HU). Male Sprague Dawley rats (8 weeks old, *n* = 6/group) were divided into ambulatory control, HU, and HU with irisin (HU + Ir, 3×/week). Lower cancellous bone volume, higher osteoclast surfaces (OcS), and lower bone formation rate (BFR) were present at the hindlimb and 4th lumbar vertebrae in the HU group while the proximal humerus of HU rats exhibited no differences in bone volume, but higher BFR and lower OcS vs. Con. Osteocyte tumor necrosis factor-α (TNF-α), interleukin-17 (IL-17), RANKL, and sclerostin were elevated in the cancellous bone of the distal femur of HU rats vs. Con, but lower at the proximal humerus in HU rats vs. Con. Exogenous irisin treatment increased BFR, and lowered OcS and osteocyte TNF-α, IL-17, RANKL, and sclerostin in the unloaded hindlimb of HU + Ir rats while having minimal changes in the humerus. In conclusion, there are site-specific and loading-specific alterations in osteocyte pro-inflammatory cytokines and bone turnover with the HU model of disuse bone loss, indicating a potential mechanosensory impact of osteocyte TNF-α and IL-17. Additionally, exogenous irisin significantly reduced the pro-inflammatory status of the unloaded hindlimb.

## Introduction

Bone is a highly adaptive tissue that responds to alterations in the mechanical strain environment. In disuse conditions where regular mechanical strains on bone are diminished, bone mass is lost primarily at weight-bearing sites^1,2^. For example, during long-duration spaceflight, bone loss in astronauts occurs in the weight-bearing tibia while minimal changes occur at the non-weightbearing radius^3^. In various animal models of disuse, like the hindlimb unloading (HU) model and actual spaceflight, similar disuse-induced reductions in bone occur, particularly in cancellous compartments^4–8^. Disuse-induced bone loss is typically characterized by increased bone resorption and decreased bone formation^6,8–10^.

Over the past several decades, the mechanosensory role of osteocytes has been identified as an important orchestrator of bone’s response to unloading/loading^11–14^. With dendritic processes extending out through the lacuna-canalicular spaces forming vast communication networks, osteocytes sense mechanical strains and signal to osteoblasts and osteoclasts to alter bone turnover. Mice with targeted ablation of osteocytes develop an aging-like skeletal phenotype with intracortical porosity and microfractures, but are also resistant to unloading-induced bone loss; these observations highlight the pivotal role of osteocytes both in overall skeletal health as well as in orchestrating alterations to unloading^15^.

Osteocytes produce and secrete proteins, such as sclerostin and receptor activator of nuclear factor κB ligand (RANKL), that are influenced by the mechanical loading state. Mice lacking osteocyte sclerostin, an inhibitor of bone formation, are protected from disuse-induced bone loss^16^ as are mice lacking osteocyte RANKL, an osteoclastogenesis regulator^17^. Cultured osteocytes treated with simulated unloading via the rotating wall vessel system exhibit increases in expression of SOST, the gene encoding sclerostin, and RANKL, suggesting direct effects of unloading on osteocytes^18^. Previously, we demonstrated differential responses of osteocyte proteins related to bone formation (sclerostin, insulin-like growth factor-1, and interleukin-6) in the unloaded hindlimb versus the weight-bearing forelimb of hindlimb unloaded rats^6^.

Beyond disuse-induced bone loss, many other conditions lead to declines in bone mass including systemic inflammatory conditions such as inflammatory bowel disease (IBD)^19^, rheumatoid arthritis^20^, and systemic lupus erythematosus^21^. Pro-inflammatory cytokines, like tumor necrosis factor-α (TNF-α), potently stimulate bone resorption and suppress bone formation^22,23^. Another pro-inflammatory cytokine, interleukin-17 (IL-17), also increases osteoclastogenesis and sensitizes osteoclast precursors to RANKL^24^. Osteocytes themselves release or express key pro-inflammatory cytokines based on evidence from osteocyte-like cell culture lines^25–29^, cultured human trabecular bone chips expressing osteocyte genes^30^, osteocyte mRNA expression in mice^31^, and immunohistochemical staining of osteocytes in rodents^32–34^. In conditions of chronic inflammation, osteocyte expression of pro-inflammatory cytokines is elevated^32–34^. In addition to directly impacting osteoclast and osteoblast activity, pro-inflammatory cytokines also upregulate classical osteocyte proteins controlling bone turnover including RANKL and sclerostin^22,32^, which themselves are elevated in inflammatory conditions including IBD, obesity, and periodontitis^33–37^.

Evidence is accruing of overlaps between the mechanosensory function of osteocytes and their inflammatory response. Pulsatile fluid flow to simulate mechanical strain in culture resulted in reduced expression of TNF-α in the MLO-Y4 osteocyte-like cell line as well as blunted IL-17A-induced elevations in TNF-α and RANKL^28^. In another in vitro study, MLO-Y4 cells cultured with TNF-α and interleukin-1β had reduced uptake of calcium and nitric oxide release when exposed to pulsatile fluid flow, which suggests these cytokines may impair the mechanosensory function of osteocytes^25^. How unloading vs. loading induces adaptations to osteocyte inflammatory factors is not fully understood but could directly influence bone turnover at local bone sites. Increased mechanical loading via exercise is recommended for both disuse-induced bone loss and inflammatory bone loss. Enhanced mechanical loading improving bone mass in conditions of low mechanical loads including spaceflight^38^, bedrest^39^, and HU in rodents^40–42^. Less empirical evidence is available on the impact of exercise/enhanced loading in inflammatory conditions, but some evidence points to improved bone outcomes^43,44^.

Exercise leads to alterations in loading, tissue metabolic state, and circulation of molecular factors released by contracting muscle (myokines). One myokine is irisin, originally described as a metabolic regulator stimulating the browning of adipose^45^. Recent research has explored how irisin also increases osteoblasts and cortical bone mass^46–48^ and regulates inflammation^49–53^. Previously, we discovered that exogenous irisin treatment ameliorated the inflammatory response of gut and bone in a rodent model of chronic IBD^51,52^. Another study found that administration of irisin prevented disuse-induced bone loss in HU^54^, but whether this was due to alterations of inflammatory factors has not been previously addressed.

The goals of this current project were two-fold: (1) to determine whether there are local loading or unloading-specific adaptations in inflammatory osteocyte proteins of HU rats and (2) to explore the impact of exogenous irisin to reduce the pro-inflammatory response of osteocytes in HU rats. We hypothesized the unloaded hindlimbs would have increased inflammatory osteocyte proteins concurrent with higher sclerostin and RANKL compared to ambulatory controls, while the forelimb would have no differences vs. control forelimbs in inflammatory proteins, but reduced sclerostin and RANKL. Secondly, we hypothesized that exogenous treatment with irisin would systemically reduce pro-inflammatory proteins in osteocytes, increase bone formation, and decrease bone resorption.

## Results

### Hindlimb unloaded animals gained less body weight than ambulatory controls

In the current study, all HU rats successfully completed 28 days of HU. Minimal porphyrin staining was evident in HU rats in the first 1–5 days of unloading, but disappeared by the second week of unloading. Over the 28-day period, the control group gained 37% of their starting bodyweight while the HU alone group gained 12% and the HU + Ir group gained 8% of their initial bodyweight (Table 1). Beginning at 2 weeks of HU throughout the rest of the study, body weights of both HU groups were lower than those of Control rats while both HU groups were not different from each other (see Table 1). Soleus wet weight at time of tissue collection was 3.3-fold lower in weight in both HU groups compared to Con (ANOVA *p* < 0.0001, *F* = 167.394, effect size = 0.949) confirming disuse of the hindlimbs.

**Table 1:** Average body weight measured in grams over the course of the experiment.

Group	Baseline	Week 1	Week 2	Week 3	Week 4
Con	260 ± 15	295 ± 16^a^	326 ± 19^a^	349 ± 27^a^	357 ± 26^a^
HU	272 ± 5	279 ± 10^ab^	293 ± 9^b^	300 ± 10^b^	307 ± 10^b^
HU + Ir	273 ± 7	274 ± 11^b^	284 ± 12^b^	292 ± 18^b^	297 ± 19^b^

Data represented as mean ± standard deviation. Superscript letters indicate groups (vertical three groups) not sharing the same letter are statistically different (from Tukey HSD; *p* < 0.05). No superscript letters indicate no statistical difference in the one-way ANOVA.

### HU resulted in declines in volumetric bone mineral density (vBMD) in the proximal tibia and femoral neck with increased vBMD in the proximal humerus

At the proximal tibia metaphysis, compared to control animals, HU caused lower total bone mineral content (BMC, ANOVA *p* < 0.0001, *F* = 23.829, effect size = 0.737; Table 2) and cancellous vBMD (ANOVA *p* < 0.0001, *F* = 13.441, effect size = 0.613), while total vBMD (ANOVA *p* = 0.037, *F* = 4.045, effect size = 0.322) and metaphyseal cortical vBMD (ANOVA *p* = 0.038, *F* = 3.971, effect size = 0.318) did not differ with our post hoc analysis. At the femoral neck, total BMC was lower in both HU groups vs. Con (ANOVA *p* = 0.004, *F* = 7.859, effect size = 0.480) while total vBMD was not statistically different between groups (ANOVA *p* = 0.056, *F* = 3.426). Cancellous vBMD at the femoral neck was lower in both HU groups vs. Con (ANOVA *p* = 0.001, *F* = 11.370, effect size = 0.572), while there were not differences in metaphyseal cortical vBMD (ANOVA *p* = 0.915, *F* = 0.089). At the proximal humerus, there were not statistical differences across groups in total BMC (ANOVA *p* = 0.057, *F* = 3.415; Table 2), but total vBMD did have statistical difference (ANOVA *p* = 0.001, *F* = 9.862, effect size = 0.537) with HU + Ir higher than Con. Both HU groups exhibited lower cancellous vBMD values at the proximal humerus (ANOVA *p* = 0.001, *F* = 11.164, effect size = 0.586), while metaphyseal cortical vBMD was higher in both HU groups vs. control (ANOVA *p* < 0.0001, *F* = 44.270, effect size = 0.839; Table 2).

**Table 2:** Peripheral quantitative computed tomographic measures of volumetric bone mineral density (vBMD) of the proximal tibia metaphysis, femoral neck, and proximal humerus.

Group	Total BMC (g)	Total vBMD (mg/cm^3^)	Cancellous vBMD (mg/cm^3^)	Metaphyseal cortical vBMD (mg/cm^3^)
*Proximal tibia*				
Con	8.7 ± 0.9^a^	448.16 ± 25.6	209.97 ± 45.6^a^	463.31 ± 21.9
HU	6.6 ± 0.3^b^	408.76 ± 32.6	128.09 ± 13.4^b^	426.32 ± 30.5
HU + Ir	6.7 ± 0.2^b^	408.07 ± 30.8	144.36 ± 23.9^b^	426.20 ± 31.3
*Femoral neck*				
Con	3.9 ± 0.4^a^	959.51 ± 47.2	667.42 ± 134.6^a^	1133.84 ± 75.2
HU	3.6 ± 0.2^b^	901.35 ± 31.3	474.25 ± 23.1^b^	1145.21 ± 18.8
HU + Ir	3.3 ± 0.1^b^	940.19 ± 47.1	496.71 ± 24.4^b^	1140.54 ± 38.6
*Proximal humerus*				
Con	5.3 ± 0.4	472.71 ± 31.4^b^	249.61 ± 24.6^a^	1094.63 ± 21.1^b^
HU	4.9 ± 0.1	500.79 ± 16.5^ab^	204.91 ± 16.7^b^	1172.05 ± 14.1^a^
HU + Ir	4.9 ± 0.2	525.85 ± 8.3^a^	211.98 ± 12.2^b^	1177.49 ± 18.5^a^

Data represented as mean ± standard deviation. Superscript letters indicate groups (vertical three groups) not sharing the same letter are statistically different (from Tukey HSD; *p* < 0.05). No superscript letters indicate no statistical difference in the one-way ANOVA.

### Cancellous bone volume was lower in HU groups in hindlimb and vertebral sites

**Fig. 1:**
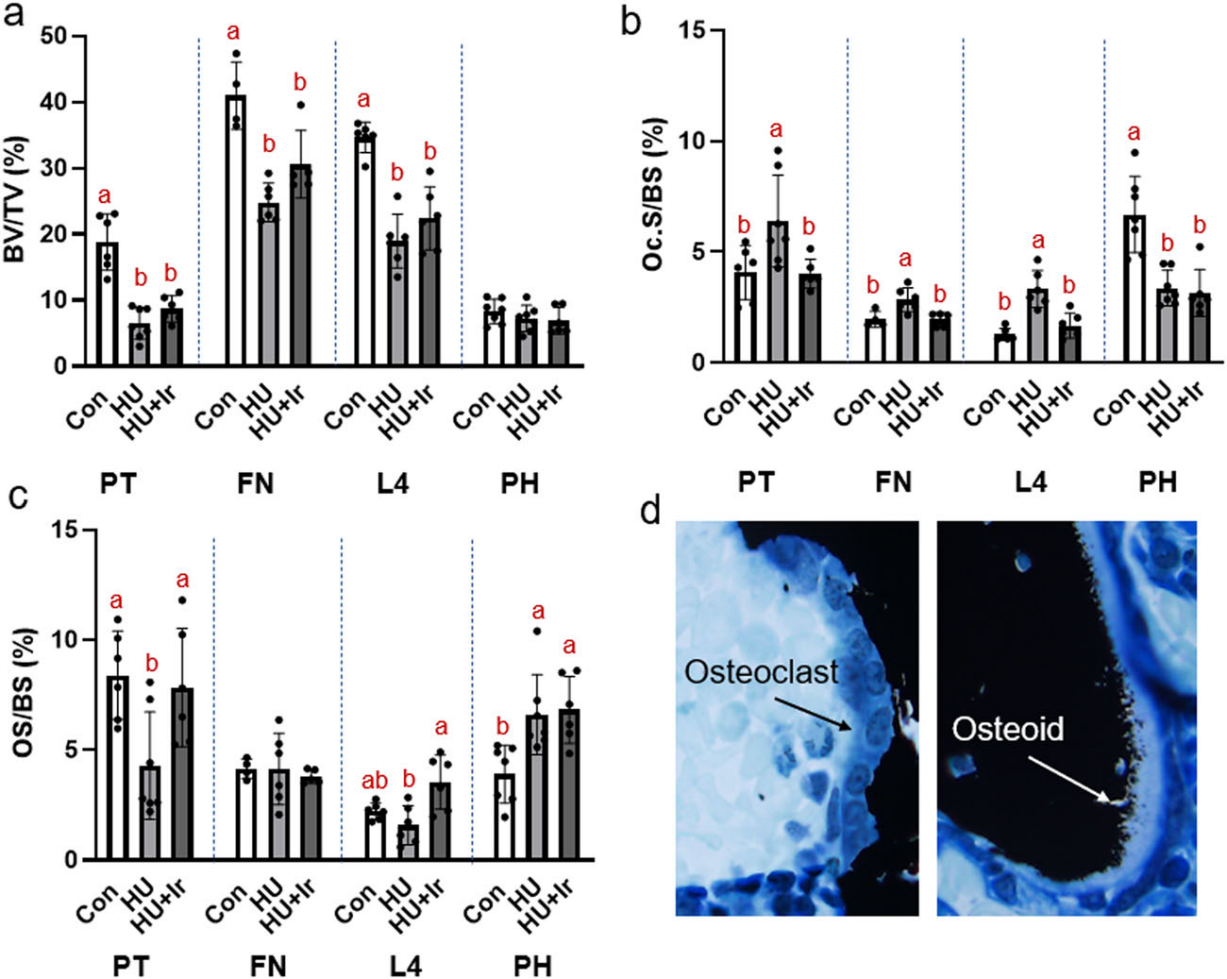
Cancellous static histomorphometry of the proximal tibia metaphysis (PT), femoral neck (FN), fourth lumbar vertebrae (L4), and proximal humerus (PH). **a** Cancellous bone volume was lower in both HU groups compared to Con at the proximal tibia, femoral neck, and L4. There were not differences in cancellous bone volume at the proximal humerus. **b** Osteoclast-covered surfaces were higher in the HU group at the proximal tibia, femoral neck, and L4 compared to Con and HU + Ir. At the proximal humerus osteoclast-covered surfaces were higher in Con compared to both HU groups. **c** Osteoid-covered surfaces were lower in HU compared to both other groups at the proximal tibia, no differences at the femoral neck, higher in HU + Ir compared to both other groups at L4, and lower in Con compared to both HU groups at the proximal humerus. **d** Representative images of osteoclast-covered surfaces and osteoid covered surfaces. Error bars = standard deviation. Groups not sharing the same letter are statistically different within that bone site (from Tukey HSD; *p* < 0.05). No letters within a site indicates no statistical differences.

For cancellous BV/TV there were statistical differences at the proximal tibia (ANOVA *p* < 0.0001, *F* = 29.871, effect size = 0.789), femoral neck (ANOVA *p* < 0.0001, *F* = 16.877, effect size = 0.738), and fourth lumbar vertebrae (ANOVA *p* < 0.0001, *F* = 27.376, effect size = 0.785). %BV/TV measured via histomorphometry was lower in both HU groups vs. Con at all sites (Fig. 1a). At the proximal humerus there were not differences among groups in %BV/TV (ANOVA *p* = 0.420, *F* = 0.913; Fig. 1a).

### Cancellous osteoclast surfaces were elevated due to HU in hindlimb and vertebral sites and lower due to HU at the humerus. Irisin treatment lowered osteoclast surfaces at those bone sites equivalent to control levels

One-way ANOVA showed statistical differences at the proximal tibia (ANOVA *p* = 0.014, *F* = 5.590, effect size = 0.411), femoral neck (ANOVA *p* = 0.007, *F* = 7.836, effect size = 0.566), and L4 (ANOVA *p* < 0.0001, *F* = 19.722, effect size = 0.724). At these three sites, osteoclast surfaces were higher in HU vs. Con and HU + Ir (Fig. 1b). At all these three sites, osteoclast surfaces for HU + Ir was not different from that value in Con animals. At the proximal humerus, osteoclast surfaces were lower in both HU groups vs. Con (ANOVA *p* < 0.0001, *F* = 16.687, effect size = 0.663; Fig. 1b).

### Cancellous osteoid surfaces were lower due to HU at the tibia and higher due to HU at the humerus. Irisin treatment in HU resulted in higher osteoid surface at the proximal tibia and fourth lumbar vertebrae

At the proximal tibia, osteoid-covered surfaces were lower in HU vs. Con and HU + Ir (ANOVA *p* = 0.013, *F* = 5.721, effect size = 0.417). At the femoral neck, there were not differences among groups (ANOVA *p* = 0.846, *F* = 0.169). At L4, HU + Ir had higher osteoid surface than HU alone (ANOVA *p* = 0.006, *F* = 7.322, effect size = 0.494). At the proximal humerus, both HU groups had higher osteoid surface than Con (ANOVA *p* = 0.005, *F* = 7.287, effect size = 0.462; Fig. 1c).

### HU resulted in lower bone formation rate (BFR) in the hindlimb and L4, but higher BFR vs. Con in the humerus. Irisin treatment increased BFR at all bone sites

**Fig. 2:**
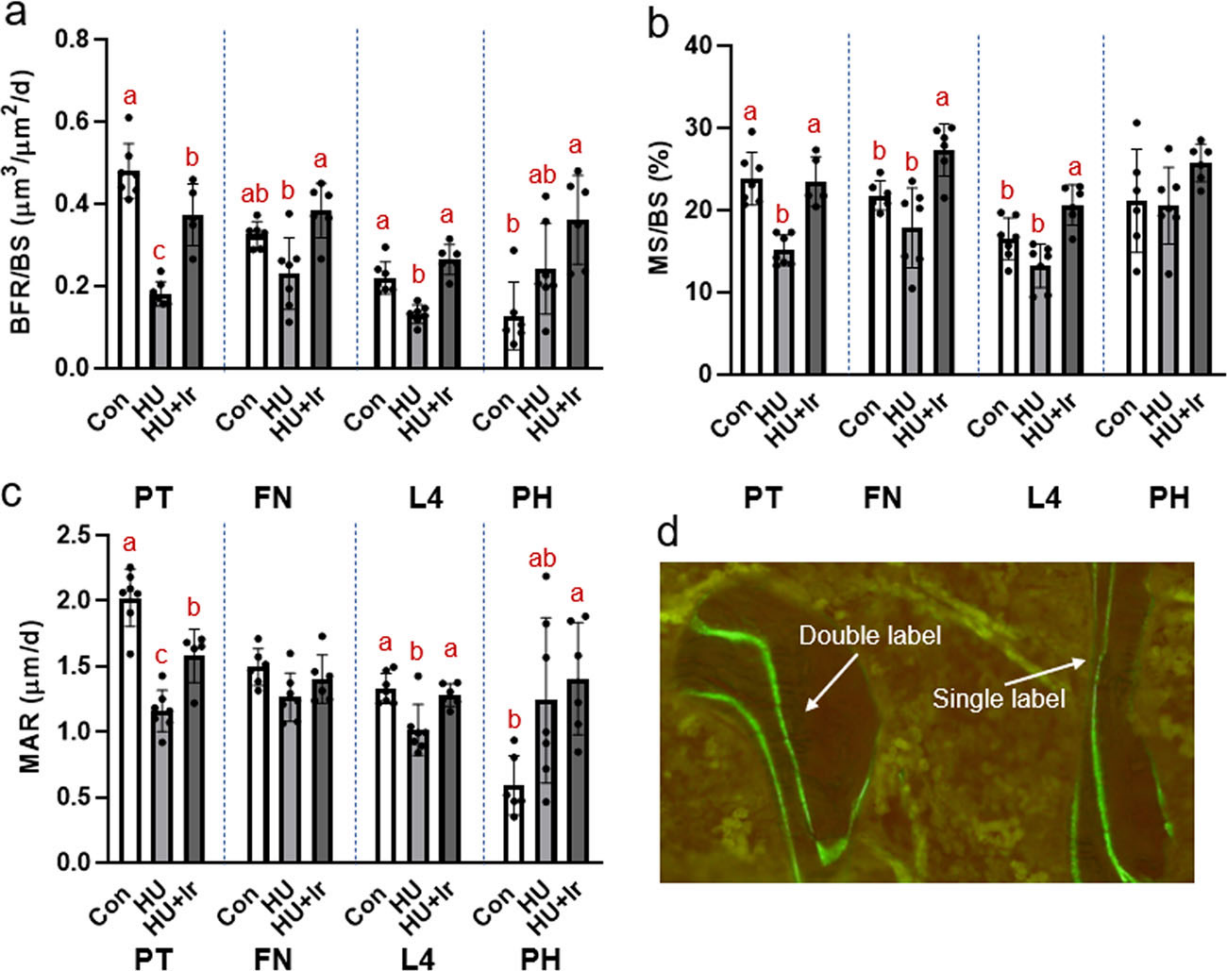
Cancellous bone formation rate of the proximal tibia metaphysis (PT), femoral neck (FN), 4th lumbar vertebrae (L4), and proximal humerus (PH). **a** At the proximal tibia, BFR was lower in HU vs. both Con and HU + Ir with HU + Ir also lower than Con. At the femoral neck, HU + Ir had higher BFR than both Con and HU. At L4, HU was lower than Con with HU + Ir higher than both groups. At the proximal humerus, Con was lower than HU + Ir with HU not different from either group. **b** Mineralized surface at the proximal tibia was lower in HU compared to both other groups with higher mineralized surface in HU + Ir compared to Con and HU at the femoral neck. At L4, mineralized surface was lower in HU compared to Con with HU + Ir higher than both HU and Con. There were not statistical differences in mineralized surface at the proximal humerus. **c** At the proximal humerus, mineral apposition rate was lower in HU compared to Con and HU + Ir with HU + Ir higher than HU alone. There were not differences at the femoral neck. At L4, mineral apposition rate was lower in HU vs. both other groups and in the proximal humerus mineral apposition rate was lower in Con vs. both HU groups. **d** Representative image of single and double label in dynamic histomorphometry for bone formation rate. Error bars = standard deviation. Groups not sharing the same letter are statistically different within that bone site (from Tukey HSD; *p* < 0.05). No letters within a site indicates no statistical differences.

At the proximal tibia, BFR was lower in HU vs. Con while HU + Ir was higher than HU alone (ANOVA *p* < 0.0001, *F* = 46.582, effect size = 0.853; Fig. 2a). Mineral apposition rate (MAR) at the proximal tibia showed the same changes as in BFR (ANOVA *p* < 0.0001, *F* = 34.804, effect size = 0.813; Fig. 2c) while mineralizing surface was lower in HU vs. Con while HU + Ir was not different from Con (ANOVA *p* < 0.0001, *F* = 21.994, effect size = 0.733; Fig. 2b). At the femoral neck, BFR was lower in HU vs. HU + Ir with Con not different from either group (ANOVA *p* = 0.003, *F* = 8.605, effect size = 0.518; Fig. 2a). For mineralizing surface at the femoral neck, HU + Ir was higher than both Con and HU (ANOVA *p* = 0.001, *F* = 11.159, effect size = 0.582; Fig. 2b) while there were not statistical differences in MAR (ANOVA *p* = 0.081, *F* = 2.954; Fig. 2c). At L4, BFR was lower in HU vs. Con and HU + Ir (ANOVA *p* < 0.0001, *F* = 27.189, effect size=0.762; Fig. 2a). In mineralizing surface, HU + Ir was higher than both Con and HU (ANOVA *p* < 0.0001, *F* = 13.750, effect size = 0.618; Fig. 2b). For MAR, HU was lower than both Con and HU + Ir (ANOVA *p* = 0.001, *F* = 9.816, effect size = 0.536; Fig. 2c). At the proximal humerus, BFR was higher in HU + Ir vs. Con with HU not different from either group (ANOVA *p* = 0.004, *F* = 7.947, effect size = 0.498; Fig. 2a). At this site, there were not differences in mineralizing surface (ANOVA *p* = 0.133, *F* = 2.299; Fig. 2b) while in MAR HU + Ir was higher than Con (ANOVA *p* = 0.02, *F* = 5.044, effect size = 0.387; Fig. 2c).

### Cathepsin K-positive-covered bone surfaces (BSs) were higher in the hindlimb of unloaded rats and the forelimb of control rats with irisin significantly lowering cathepsin-K in both locations

**Fig. 3:**
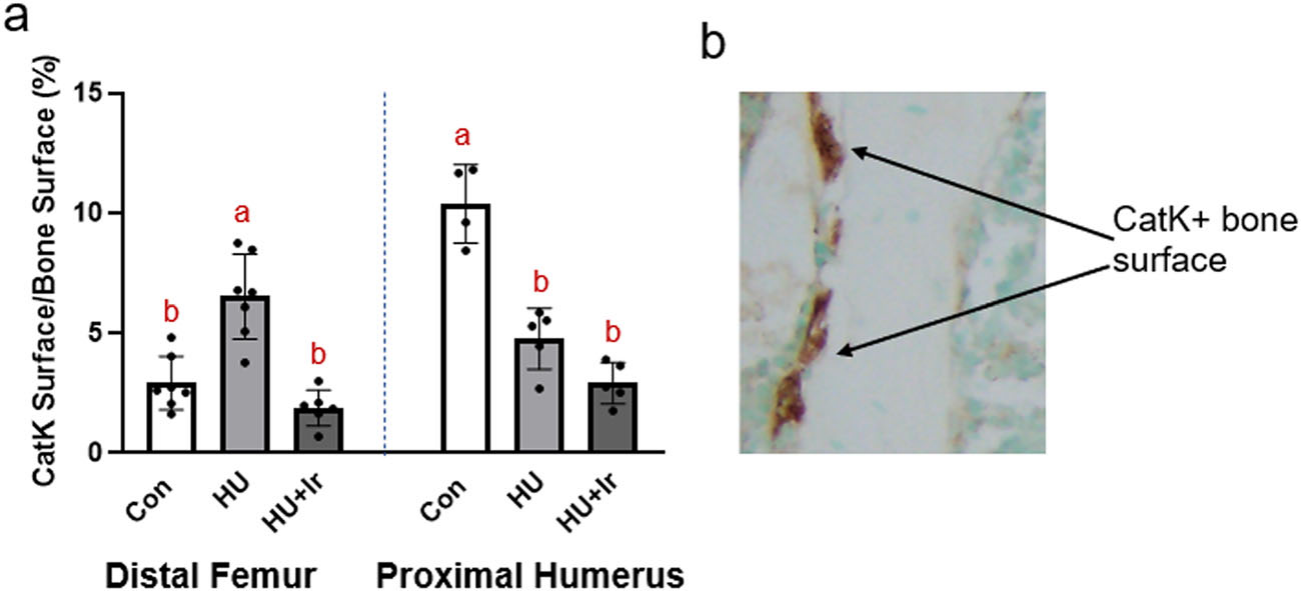
Immunohistochemistry for cathepsin-K-covered bone surfaces, a surrogate of osteoclast surfaces, at the distal femur and proximal humerus. **a**) %Cathepsin-K-positive surfaces were higher in the distal femur of HU rats vs. both other groups. In the proximal humerus, Con had the highest %Cathepsin-K-positive surfaces followed by HU and then HU + Ir. Error bars = standard deviation. Groups not sharing the same letter are statistically different within that bone site (from Tukey HSD; *p* < 0.05). **b**) Representative histological images of cathepsin-K staining in cancellous bone.

At the distal femur, %Cathepsin-K-positive BSs, to measure osteoclast surfaces, were higher in HU vs. both Con and HU + Ir (ANOVA *p* < 0.0001, *F* = 23.173, effect size = 0.732; Fig. 3). At the proximal humerus, %Cathepsin-K-positive surfaces were highest in Con vs. both HU groups (ANOVA *p* < 0.0001, *F* = 41.118, effect size = 0.882; Fig. 3).

### Osteocyte TNF-α and IL-17 were higher in the hindlimb of unloaded rats and the forelimb of control rats. Irisin-treatment reduced osteocyte TNF-α and IL-17 at the distal femur

**Fig. 4:**
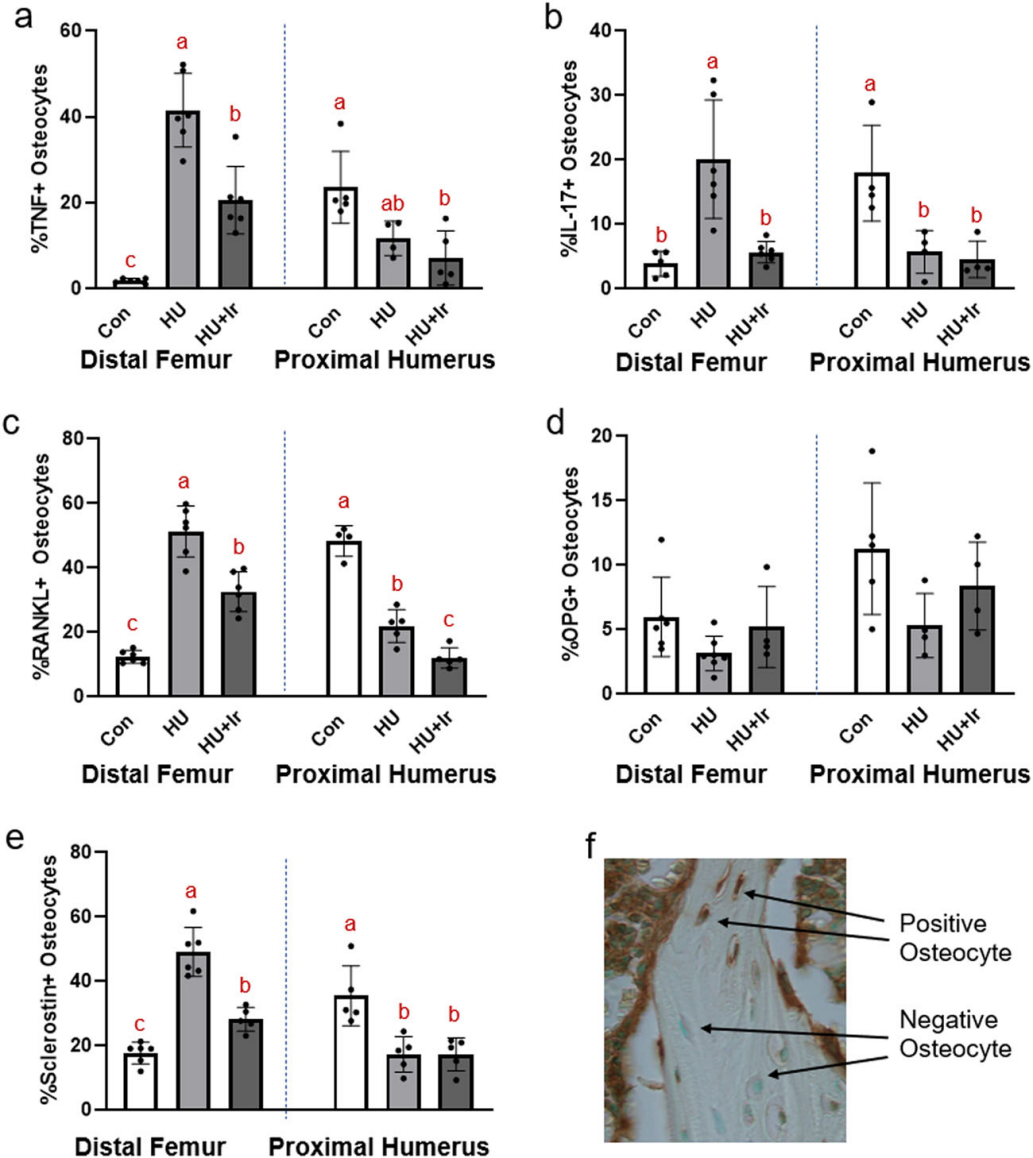
Immunohistochemistry of osteocyte proteins in the cancellous bone of the distal femur and proximal humerus. **a** %TNF-α-positive osteocytes were higher in HU compared to both Con and HU + Ir with HU + Ir higher than Con. At the proximal humerus, Con had higher %TNF-α-positive osteocytes compared to both HU groups. **b** %IL-17-positive osteocytes were higher in HU vs. Con and HU + Ir at the distal femur and higher in Con vs. both HU groups at the proximal humerus. **c** %RANKL-positive osteocytes were higher in HU vs. Con and HU + Ir with HU + Ir higher than Con at the distal femur. At the proximal humerus, Con was higher than HU and HU was higher than HU + Ir. **d** There were not statistical differences in %OPG-positive osteocytes at either bone site. **e** %Sclerostin-positive osteocytes were higher in HU vs. Con and HU + Ir with HU + Ir higher than Con at the distal femur. At the proximal humerus, %sclerostin-positive osteocytes were higher in Con vs. both HU groups. **f** Representative image of osteocyte immunohistochemistry. Error bars = standard deviation. Groups not sharing the same letter are statistically different within that bone site (from Tukey HSD; *p* < 0.05).

At the distal femur cancellous bone, TNF-α-positive osteocytes were highest in HU, followed by HU + Ir and Con (ANOVA *p* < 0.0001, *F* = 52.199, effect size = 0.874; Fig. 4a). IL-17-positive osteocytes at the distal femur were higher in HU vs. Con and HU + Ir (ANOVA *p* < 0.0001, *F* = 14.077, effect size = 0.668; Fig. 4b). At the proximal humerus, TNF-α-positive osteocytes (ANOVA *p* = 0.007, *F* = 8.014, effect size = 0.593) were higher in Con vs. HU + Ir. IL-17-positive osteocytes at the proximal humerus were lower in both HU groups vs. Con animals (ANOVA *p* = 0.008, *F* = 8.830, effect size = 0.662; Fig. 4a, b).

### RANKL-positive osteocytes were higher in HU hindlimbs and control forelimbs with irisin-treatment resulting in declines in RANKL at both sites

At the distal femur, RANKL-positive osteocytes were highest in HU with the group average of HU + Ir lower than HU, but higher than Con (ANOVA *p* < 0.0001, *F* = 64.871, effect size = 0.896; Fig. 4c). At the proximal humerus, both HU groups were lower than Con with HU + Ir lower than HU alone (ANOVA *p* < 0.0001, *F* = 78.287, effect size = 0.934; Fig. 4c). OPG-positive osteocytes were not different at either bone sites (ANOVA *p* = 0.146*, F* = 2.218 for distal femur, *p* = 0.130, *F* = 2.523 for proximal humerus; Fig. 4d).

### Sclerostin-positive osteocytes were higher in the hindlimb of the HU group and forelimb of the control group with irisin significantly lowering sclerostin in the hindlimb of the HU group

At the distal femur, sclerostin-positive osteocytes were higher in both HU groups vs. Con with HU + Ir lower than HU alone (ANOVA *p* < 0.0001, *F* = 54.154, effect size = 0.886). At the proximal humerus, sclerostin-positive osteocytes were lower in both HU groups vs Con (ANOVA *p* = 0.002, *F* = 11.531, effect size = 0.658; Fig. 4e).

## Discussion

The primary findings of this study are (1) HU results in site-specific alterations in bone turnover with increased bone resorption and decreased bone formation in vertebral and hindlimb bone, with an opposite response in the forelimb based on the loading/unloading condition, (2) osteocyte inflammatory proteins (TNF-α, IL-17) were elevated in the HU hindlimbs, but lower versus controls in the HU forelimbs, indicating site-specific and loading-specific adaptations in osteocyte inflammatory response in conjunction with altered bone resorption and formation, and (3) treatment with exogenous irisin prevented the increased osteocyte inflammatory response in the unloaded hindlimb while having minimal impact in the loaded forelimb.

HU is a well-accepted animal model to characterize disuse-induced bone loss and simulate spaceflight. Losses of 15–25% of cancellous BV/TV in hindlimb metaphyseal sites are consistently observed after 21–28 days of HU in rodents^4–7^. In this current study, young male rats had 9% lower total vBMD and 39% lower cancellous vBMD at the proximal tibia metaphysis compared to ambulatory controls. At the femoral neck, similar changes were seen with 6% lower total vBMD and 28% lower cancellous vBMD in HU rats vs. ambulatory controls. Cancellous %BV/TV, measured via histomorphometry, was 3-fold lower in HU rats at the proximal tibia, 44% lower at the 4th lumbar vertebra, and 39% lower at the femoral neck. Although the weight-bearing proximal humerus of HU rats had 6% greater total vBMD than the humerus of ambulatory rats, cancellous vBMD at this bone site was 18% lower and cancellous bone volume non-statistically 12% lower in HU rats vs. ambulatory controls. We are uncertain of why cancellous bone at the humeral metaphysis would be selectively affected in rats subjected to HU. Perhaps there were modest alterations in focal strain distributions in the forelimbs due to postural/loading differences with HU. The higher total vBMD and metaphyseal cortical vBMD in HU (7% higher vs. Con) at the proximal humerus suggest enhanced loading of the forelimb during HU. In sum, our data confirm disuse-induced cancellous bone loss at the proximal tibia, 4th lumbar vertebrae, and femoral neck, and higher total and cortical bone mass at the proximal humerus after 28 days of HU.

Multiple previous investigations in humans and in rodents have demonstrated increased bone resorption and decreased bone formation with disuse^6,8–10^. In our young male rats, osteoclast-covered surfaces were 57% higher at the proximal tibia, 163% higher at L4, and 89% higher at the femoral neck in HU rats vs. the same site in ambulatory controls. BFR was 62% lower at the proximal tibia, 41% lower at L4, and 28% lower at the femoral neck in HU animals vs. ambulatory controls. In contrast, cancellous bone osteoclast-covered surfaces at the proximal humerus of HU rats were 42% lower than those of ambulatory controls and BFR was (non-statistically different) 2-fold higher in the HU proximal humerus compared to the ambulatory animals. Previously, we demonstrated 48% increased osterix-covered surfaces and 53% decreased cathepsin-K-covered surfaces in the loaded humerus of skeletally mature HU rats as surrogate measures of altered osteoblast and osteoclast activity, respectively^6^. These current data corroborate and broaden that previous data more definitively demonstrating opposite changes in bone cell activity in the hindlimb vs. the forelimb of HU rats. We hypothesize these differences are due to local changes in the mechanical strain environment, with the hindlimb and vertebral bone in mechanical unloading while the forelimbs experience enhanced strain or altered strain distribution.

Osteocytic proteins known to control bone turnover, like RANKL and sclerostin, have been implicated as major orchestrators of disuse-induced bone loss, with increased RANKL and sclerostin in conditions of unloading^17,55^. Cultured osteocytes exposed to oscillatory fluid flow exhibit decreased production of RANKL^14^ and regions of loaded ulnar bone with the most strain had the greatest reduction in sclerostin^55^. Our current study demonstrates 4-fold higher cancellous RANKL-positive osteocytes and nearly 3-fold higher sclerostin-positive osteocytes in the HU distal femur compared to ambulatory controls. In contrast, the proximal humerus cancellous bone of HU rats had 2-fold lower RANKL-positive osteocytes and sclerostin-positive osteocytes compared to the same bone site in ambulatory controls. These changes in RANKL and sclerostin correspond with changes in % osteoclast surface and BFR at both sites and further indicate localized adaptations to loads within the same animal.

Pro-inflammatory cytokines TNF-α and IL-17 are both potent stimulators of osteoclasts and synergize with RANKL to increase osteoclastogenesis^22,24,56,57^. Previously, we identified increased osteocyte pro-inflammatory cytokines in age-matched ambulatory rats with systemic inflammation^51,52^. In this study, we aimed to determine if these factors were altered in HU. TNF-α-positive osteocytes were nearly 23-fold higher in the distal femur of HU rats vs. controls and IL-17-positive osteocytes were elevated 5-fold compared to control. Contrary to our original hypothesis, the response in the humerus of HU rats was the opposite of the femur with 2-fold lower TNF-α-positive osteocytes and 3-fold lower IL-17-positive osteocytes compared to those in the humerus of ambulatory control rats. While these results did not match our original hypothesis, they are consistent with the alterations in bone turnover seen at both bone sites with higher IL-17 and TNF-α associated with higher % osteoclast surfaces and lower bone formation. Since pro-inflammatory factors like TNF-α stimulate RANKL and sclerostin^22,32^, the responses in these osteocytic proteins correspond with the changes in inflammatory proteins. Our measures also match those of in vitro studies exposing osteocytes to increased mechanical loading via pulsatile fluid flow, resulting in alterations of osteocyte pro-inflammatory cytokines and RANKL^28,30,58^. For example, cultured primary human osteocytes exposed to serum from rheumatoid arthritis patients had increased gene expression of RANKL and decreased expression of OPG while pulsatile fluid flow decreased the ratio of RANKL/OPG increases^59^. Therefore, we hypothesize the alterations in osteocyte pro-inflammatory cytokines are related to the loading state of the bone and these pro-inflammatory factors may be involved in orchestrating the changes in RANKL, sclerostin, and bone turnover.

Previously, we and others have identified a potential anti-inflammatory role of the myokine irisin^49–53^. In animal models of IBD, exogenous treatment with irisin successfully mitigated the increase in osteocyte pro-inflammatory cytokines, while lowering osteoclast surfaces and increasing BFR^51,52^. One previously published study demonstrated prevention of bone loss in hindlimb unloaded mice with a dose of recombinant irisin 1000× higher than that of this study^54^, but did not investigate the impact on inflammatory markers. In the current study, we discovered that treatment with exogenous irisin lowered TNF-α-positive osteocytes by 50% and IL-17-positive osteocytes 4-fold in the unloaded distal femur concurrent with 37% lower RANKL-positive osteocytes and 42% lower sclerostin-positive osteocytes. Irisin treatment resulted in a 2-fold increase in BFR at the proximal tibia and L4 and a 65% higher BFR at the femoral neck. At L4 and the femoral neck, the irisin-treated group had BFR levels not different from ambulatory controls, while at the proximal tibia BFR was higher than HU alone, but still 21% lower than ambulatory controls. At all hindlimb bone and vertebral sites, osteoclast surfaces in irisin-treated HU rats were not different from ambulatory controls. With higher BFR and lower osteoclast surfaces due to exogenous irisin treatment, we hypothesize these changes are due in part to the impact of irisin treatment on osteocyte proteins. Therefore, while the dose was unable to mitigate loss of bone mass, exogenous irisin treatment did mitigate the pro-inflammatory response and had a favorable impact on osteoblast activity.

Originally, we hypothesized that exogenous irisin would have a systemic impact regardless of bone site in the HU model; however, we found inflammatory proteins in humeral osteocytes of irisin-treated HU rats exhibited minimal differences vs. the same loaded bone site in untreated HU rats. While there were significant differences in osteoclast surfaces, IL-17-positive osteocytes, and sclerostin-positive osteocytes in the humerus of HU rats vs. the humerus of control rats, these same factors were not different in irisin-treated HU rats vs. untreated HU. The only differences between irisin-treated and untreated HU rat humeri were 50% higher BFR compared to HU alone (not statistically different) and 42% lower RANKL-positive osteocytes. Since bone turnover and osteocyte protein measures in the forelimbs vs. the hindlimbs of HU rats suggest altered loading responses, we speculate these difference in response to irisin may be influenced by the loading state of the bone; however, this is only speculation as multiple other factors that we could not address with this study could influence these site-specific differences. Further research will need to address the interaction of exogenous irisin treatment and loading to determine if there is an interaction with mechanical signals in bone and the effects of irisin treatment.

Treatments for disuse-induced bone loss often aim to prevent bone loss through interventions that increase mechanical loading on bone. In animal models, jump training and simulated resistance training improve bone outcomes^40,42^. Much lower levels of mechanical stimulation can also have an impact. Only 10 min per day of low-level mechanical stimulation via an oscillating platform normalizes BFR to levels seen in control animals^41^ and 75 min of normal weightbearing per day in hindlimb unloaded mice attenuates disuse-induced loss of cancellous bone^60^. Whether interventions like these alter local pro-inflammatory osteocyte proteins has not yet been examined. Several therapeutics that influence inflammatory status without altering mechanical loading exert positive impacts on bone during disuse, including resveratrol^61^ and high doses of vitamin E^62^. Future research needs to investigate the potential for anti-inflammatory treatments to alter inflammatory signaling in osteocytes with the aim of protecting against disuse bone loss, either alone or in combination with other anti-resorptive or anabolic bone therapies.

One important consideration of this current work is the use of young, growing rats. In our previous work utilizing skeletally mature rats (7 months old), cancellous bone volume at the proximal tibia was 24% lower after 28 days of HU versus that in ambulatory controls^6^, while the same period of HU resulted in a 65% lower cancellous bone volume at the same bone site in young rats (3 months old), indicating a nearly 3-fold larger response to unloading in younger animals. Importantly, we found no differences in osteocyte TNF-α between the hindlimb and forelimb of skeletally mature HU rats^6^, while we found significant differences in this same measure in unloaded vs. loaded bone in young HU rats. Others have documented diminished responsiveness of bone to altered loading with age^63^, but differences in the local pro-inflammatory response have not been addressed, to our knowledge. The data from our two different studies indicate potential age-related differences in inflammatory signaling in osteocytes, which warrants further investigation.

Limitations of our study include the collection of bone tissue at only one timepoint after four continuous weeks of HU. We, therefore, do not know the time course of changes in osteocyte proteins over the period of disuse nor the impact of irisin treatment at various timepoints. Additionally, we only utilized one dose of irisin that we previously determined mitigated inflammatory responses in chronic models of IBD model^51,52^. We also did not incorporate ambulatory irisin only controls, but based on our previous work (utilizing animals of the same age, sex, strain, and diet), we did not find significant differences in irisin treatment in the ambulatory controls^51,52^. Future research should address the impact of irisin with enhanced loading on bone. The irisin dose utilized in this study, while able to mitigate inflammatory changes, did not prevent/mitigate bone loss due to HU. Based on the alterations in inflammatory proteins and bone turnover markers, we hypothesize a higher dose or different dosing regimen could prevent bone loss, as previously described in a mouse model with different irisin dosing^54^. While we believe this in vivo model utilizing the forelimb and hindlimb of HU rats leads to unique data regarding the local inflammatory response of osteocytes to loading/unloading, other studies with controlled external loading could build on this work to validate this response, as well as to determine the level and frequency of loading needed to elicit changes in osteocyte pro-inflammatory factors.

In conclusion, this study comparing responses in forelimb and hindlimb of hindlimb unloaded rats demonstrates loading-specific adaptations of pro-inflammatory osteocyte proteins, matching changes seen in well-studied mechanosensitive osteocyte proteins, sclerostin and RANKL. Changes in osteocyte proteins corresponded with site-specific changes in bone turnover. Therefore, osteocyte inflammatory proteins may be partly mechanically regulated and upregulation of these factors in disuse could contribute to disuse-induced bone loss. In addition, exogenous treatment with irisin mitigated inflammatory changes in the unloaded bone but had minimal effects in the loaded forelimb, indicating irisin may mimic the anti-inflammatory effect of enhanced loading in a state of mechanical unloading. Further research should address both inflammatory osteocyte proteins and their responsiveness to the mechanical loading environment within bone as well as the anti-inflammatory effect of irisin in disuse-induced bone loss.

## Methods

### Animals

Eighteen Sprague-Dawley rats (male, 6 weeks old) were ordered from Envigo (Houston, TX) and singly housed in an institutionally approved animal facility with 12-h light–dark cycles. At 7 weeks of age, rodents were switched from the standard rodent chow of the animal facility (Teklad 2018; Envigo, Houston, TX, USA) to the purified AIN93G chow for growing rodents (Research Diets, Inc., New Brunswick, NJ, USA). The age of animals and the diet were utilized to compare to previous work with age-matched and diet-matched groups^51,52^. At 8 weeks of age, animals were randomly divided into three different groups (*n* = 6/group): ambulatory cage control (Con), hindlimb unloaded (HU), and hindlimb unloaded with exogenous irisin treatment (HU + Ir). The irisin-treated HU group was given injections of recombinant irisin (Adipogen, San Diego, CA, USA) intraperitoneally 3×/week as previously described^51,52^. HU was maintained continuously for 28 days. Fluorochrome calcein labels (Sigma Aldrich, St Louis, MO, USA) were injected intraperitoneally 8 and 3 days prior to termination to label mineralized surfaces on bone. Following 4 weeks of HU, animals were euthanized and tissues were collected. All animal procedures were approved by the Texas A&M Institutional Animal Use and Care Committee and conform to the NIH Guide for the Care and Use of Laboratory Animals.

### Hindlimb unloading

HU was achieved by tail suspension based on the methods developed by Morey-Holton et al^64^. as previously described^6^. Briefly, rats were anesthetized via inhaled vaporized isoflurane and tails were cleaned before application of a custom harness to the lateral sides of the tail via a thin layer of adhesive (Amazing Goop, Eclectic Products, Los Angeles, CA, USA). After the harness was dry, rats were then placed individually in a 46 × 46 × 46 cm cage with a pulley system at the top. After 6 h of acclimation to the new cage and food and water system and to ensure the harness was completely dry, the harness was lifted on the pulley system to suspend the hindlimbs off the ground at approximately a 30° head down tilt. The forelimbs remained fully weight-bearing allowing the rat to ambulate on the cage floor. *Ad libitum* access to food and water was maintained throughout the entire suspension period. Every 12 h throughout the 28-day period the health of the rats was monitored and the suspension height was adjusted, if needed, to ensure hindlimbs remained elevated off the cage floor. Any health check during the animals’ dark cycle was completed with red light. During the unloading period rats had access to environmental enrichment devices (chew toys and paper towels) and minimal bedding. Soleus muscle was isolated and weighed at the time of tissue collection to validate the efficacy of hindlimb disuse.

### Peripheral quantitative computed tomography

Ex vivo pQCT scans of the proximal tibia metaphysis, right femoral neck, and proximal humerus were completed on a Stratec XCT Research-M device (Norland Corp., Fort Atkinson, WI, USA). Metaphyseal BMC and vBMD was measured at the proximal tibia and proximal humerus from four slices located ~1 mm distal to the growth plate. Three contiguous slices were averaged to provide one value for each variable at these two sites. Three scans of the femoral neck were averaged together for each variable. Scans were completed at 2.5 mm/s scan speed, 100 μm voxel resolution, and 0.5 mm slice spacing.

### Dynamic and static cancellous histomorphometry

For cancellous histomorphometry measures, undemineralized left proximal tibia, 4th lumbar vertebra, left proximal femur, and left proximal humerus were fixed in 4% phosphate-buffered formalin for 24 h and then subjected to serial dehydration and embedded in methyl methacrylate (J.T. Baker, VWR, Radnor, PA, USA). Serial frontal sections were cut 8 μm-thick and left unstained for fluorochrome calcein label measurements as previously described^33,34,51,52^. Histomorphometric analyses were performed using OsteoMeasure Analysis System, version 3.3 (OsteoMetrics, Inc., Atlanta, GA, USA). For the proximal tibia, a region of interest (ROI) of ~8 mm^2^ at ×20 magnification was analyzed while ROI’s of ~3 mm^2^ were used for L4 and the femoral neck and ~4 mm^2^ for the proximal humerus. At all sites, endocortical edges and primary spongiosa were not included in the ROI. Total BS, single-labeled surface (sLS/BS), double-labeled surface (dLS/BS), mineralized surface (MS/BS), and interlabel distances were measured at ×20 objective. MAR was calculated from the interlabel distance and the days between label administration. Bone formation rate (BFR/BS) was determined by multiplying MS/BS by MAR. Additionally, 4 μm-thick sections were treated with von Kossa stain and tetrachrome counterstain in order to measure cancellous bone volume as a percent of total tissue volume (BV/TV) and osteoid (OS/BS) and osteoclast (Oc.S/BS) surfaces as a percent of total cancellous surface measured at ×40 objective. All analyses were completed by the same individual. All nomenclature for cancellous histomorphometry follows standard usage^65^.

### Bone immunohistochemistry

Left distal femurs and right proximal humeri were fixed in 4% phosphate-buffered formalin for 24 h at 4 °C and then decalcified in a sodium citrate/formic acid solution for ~14 days then stored in 70% ethanol. Sections were then further dehydrated in Thermo-Scientific STP 120 Spin Tissue Processor, paraffinized via a Thermo Shandon Histocenter 3 Embedding tool, sectioned to ~6 µm thickness, mounted on positively charged slides, and immunostained using an avidin–biotin method as previously described^6,33,34,51,52^. Samples were incubated with the following primary antibodies: polyclonal rabbit anti-rat TNF-α (LifeSpan BioSciences, Inc, Seattle, WA, USA), rabbit polyclonal anti-IL-17 (Abcam), rabbit polyclonal anti-RANKL (Abcam), rabbit polyclonal anti-OPG (Biorbyt, San Francisco, CA, USA), polyclonal goat anti-mouse sclerostin (R&D Systems, Minneapolis, MN, USA), and rabbit polyclonal anti-cathepsin K (Abcam). Peroxidase development was performed with an enzyme substrate kit (DAB, Vector Laboratories). Counterstaining was conducted with methyl green (Vector Laboratories). Negative controls for all antibodies were completed by omitting the primary antibody. On each section excluding cathepsin K stains, the percentage of osteocytes staining positively for the protein was quantified within a 4 mm^2^ ROI within distal femur and proximal humerus cancellous bone. For cathepsin K, cathepsin K-positive surface were normalized to total BS within the ROI. All analyses were completed by the same individual.

### Statistical analyses

All data were tested for normality. Data were analyzed via a one-way ANOVA. All *p*-values and *F* statistics are reported are from the one-way ANOVA. Statistical significance was determined at *p* < 0.05. If the one-way ANOVA was statistically significant, effect sizes (partial eta squared) were reported, and a Tukey HSD post hoc test was completed to determine which groups differed from each other. Statistical analyses were completed on SPSS Statistics 25 (IBM; Armonk, NY, USA). All data are represented as mean ± standard deviation.

### Reporting summary

Further information on experimental design is available in the Nature Research Reporting Summary linked to this paper.

## Supplementary information

Reporting Summary Checklist FLAT

## Data Availability

The datasets generated during and/or analyzed during the current study are available from the authors on reasonable request.

## References

[1] LeBlanc AD, Spector ER, Evans HJ, Sibonga JD (2007). Skeletal responses to space flight and the bed rest analog: a review. J. Musculoskelet. Neuronal Interact..

[2] LeBlanc AD (2000). Bone mineral and lean tissue loss after long duration space flight. J. Muscoskelet. Neuronal Interact..

[3] Vico L (2000). Effects of long-term microgravity exposure on cancellous and cortical weight-bearing bones of cosmonauts. Lancet.

[4] Bloomfield SA, Allen MR, Hogan HA, Delp MD (2001). Site- and compartment-specific changes in bone with hindlimb unloading in mature adult rats. Bone.

[5] Globus RK, Bikle DD, Morey-Holton E (1986). The temporal response of bone to unloading. Endocrinology.

[6] Metzger CE (2017). Differential responses of mechanosensitive osteocyte proteins in fore- and hindlimbs of hindlimb-unloaded rats. Bone.

[7] Saxena R, Pan G, Dohm ED, McDonald JM (2011). Modeled microgravity and hindlimb unloading sensitize osteoclast precursors to RANKL-mediated osteoclastogenesis. J. Bone Min. Metab..

[8] Tavella S (2012). Bone turnover in wild type and -pleiotrophin-transgenic mice housed for three months in the International Space Station (ISS). PLoS ONE.

[9] Dehority W (1999). Bone and hormonal changes induced by skeletal unloading in the mature male rat. Am. J. Physiol..

[10] Zerwekh JE, Ruml LA, Gottschalk F, Pak CYC (1998). The effects of twelve weeks of bed rest on bone histology, biochemical markers of bone turnover, and calcium homeostasis in eleven normal subjects. J. Bone Min. Res..

[11] Bonewald LF (2011). The amazing osteocyte. J. Bone Min. Res..

[12] Bonewald LF, Johnson ML (2008). Osteocytes, mechanosensing and Wnt signaling. Bone.

[13] Burger EH, Klein-Nulend J (1999). Mechanotransduction in bone—role of the lacunocanalicular network. FASEB J..

[14] You L (2008). Osteocytes as mechanosensors in the inhibition of bone resorption due to mechanical loading. Bone.

[15] Tatsumi S (2007). Targeted ablation of osteocytes induces osteoporosis with defective mechanotransduction. Cell.

[16] Lin C (2009). Sclerostin mediates bone response to mechanical unloading through antagonizing Wnt/b-Catenin signaling. J. Bone Min. Res..

[17] Xiong J (2011). Matrix-embedded cells control osteoclast formation. Nat. Med..

[18] Spatz JM (2015). The Wnt inhibitor sclerostin is up-regulated by mechanical unloading in osteocytes in vitro. J. Biol. Chem..

[19] Agrawal M (2011). Bone, inflammation, and inflammatory bowel disease. Curr. Osteoporos. Rep..

[20] Haugeberg G, Uhlig T, Falch JA, Halse JI, Kvien TK (2000). Bone mineral density and frequency of osteoporosis in female patients with rheumatoid arthritis. Arthritis Rheum..

[21] Bultink IEM (2012). Osteoporosis and fractures in systemic lupus erthyematosus. Arthritis Care Res..

[22] Nanes MS (2003). Tumor necrosis factor-α: molecular and cellular mechanisms in skeletal pathology. Gene.

[23] Redlich K, Smolen JS (2012). Inflammatory bone loss: pathogenesis and therapeutic intervention. Nat. Rev. Drug Discov..

[24] Adamopoulos IE (2010). Interleukin-17A upregulates receptor activator of NF-κB on osteoclast precursors. Arthritis Res. Ther..

[25] Bakker AD (2009). Tumor necrosis factor-α and interleukin-1β modulate calcium and nitric oxide signaling in mechanically stimulated osteocytes. Arthritis Rheum..

[26] Chhana A (2018). Monosodium urate crystals reduce osteocyte viability and indirectly promote a shift in osteocyte function towards a proinflammatory and proresorptive state. Arthritis Res. Ther..

[27] Kanaji A (2009). Co–Cr–Mo alloy particles induce tumor necrosis factor alpha production in MLO-Y4 osteocytes: a role for osteocytes in particle-induced inflammation. Bone.

[28] Liao C (2017). Shear stress inhibits IL-17A-mediated induction of osteoclastogenesis via osteocyte pathways. Bone.

[29] Ormsby RT (2019). Osteocytes respond to particles of clinically-relevant conventional and cross-linked polyethylene and metal alloys by up-regulation of resorptive and inflammatory pathways. Acta Biomater..

[30] Pathak JL (2016). Systemic inflammation affects human osteocyte-specific protein and cytokine expression. Calcif. Tissue Int..

[31] Farr JN (2016). Identification of senescent cells in the bone microenvironment. J. Bone Min. Res..

[32] Baek K (2014). TNF-α upregulates sclerostin expression in obese mice fed a high-fat diet. Cell Physiol..

[33] Metzger CE, Narayanan SA, Zawieja DC, Bloomfield SA (2017). Inflammatory bowel disease in a rodent model alters osteocyte protein levels controlling bone turnover. J. Bone Min. Res..

[34] Metzger CE, Gong S, Aceves M, Bloomfield SA, Hook MA (2019). Osteocytes reflect a pro-inflammatory state following spinal cord injury in a rodent model. Bone.

[35] Graves DT (2018). Osteocytes play an important role in experimental periodontitis in healthy and diabetic mice through expression of RANKL. J. Clin. Periodontol..

[36] Kim JH (2017). Tumor necrosis factor-α antagonist diminishes osteocytic RANKL and sclerostin expression in diabetes rats with periodontitis. PLoS ONE.

[37] Kim JH, Lee DE, Cha JH, Bak EJ, Yoo YJ (2014). Receptor activator of nuclear factor‐κB ligand and sclerostin expression in osteocytes of alveolar bone in rats with ligature‐induced periodontitis. J. Peridontol..

[38] Smith SM (2012). Benefits for bone from resistance exercise and nutrition in long-duration spaceflight: evidence from biochemistry and densitometry. J. Bone Min. Res..

[39] Belavỳ DL (2015). Evidence for an additional effect of whole-body vibration above resistive exercise alone in preventing bone loss during prolonged bed rest. Osteoporos. Int..

[40] Ju YI (2013). Jump exercise during hindlimb unloading protect against the deterioration of trabecular bone microarchitecture in growing young rats. SpringerPlus.

[41] Rubin C, Xu G, Judex S (2001). The anabolic activity of bone tissue, suppressed by disuse, is normalized by brief exposure to extremely low-magnitude mechanical stimuli. FASEB J..

[42] Swift JM, Nilsson MI, Hogan HA, Sumner LR, Bloomfield SA (2010). Simulated resistance training during hindlimb unloading abolishes disuse bone loss and maintains muscle strength. J. Bone Min. Res..

[43] De Jong Z (2004). Slowing of bone loss in patients with rheumatoid arthritis by long-term high-intensity exercise: results of a randomized, controlled trial. Arthritis Rheum..

[44] Robinson RJ (1998). Effect of a low-impact exercise program on bone mineral density in Crohn’s disease: a randomized controlled trial. Gastroenterology.

[45] Bostrӧm P (2012). A PGC1-a-dependent myokine that drives brown-fat-like development of white fat and thermogenesis. Nature.

[46] Colaianni G (2015). The myokine irisin increases cortical bone mass. PNAS.

[47] Qiao XY (2016). Irisin promotes osteoblast proliferation and differentiation via activating the MAP kinase signaling pathways. Sci. Rep..

[48] Zhang J (2017). Exercise-induced irisin in bone and systemic irisin administration reveal new regulatory mechanisms of bone metabolism. Bone Res..

[49] Li DJ, Li YH, Yuan HB, Qu LF, Wang P (2017). The novel exercise-induced hormone irisin protects against neuronal injury via activation of the Akt and ERK1/2 signaling pathways and contributes to the neuroprotection of physical exercise in cerebral ischemia. Metabolism.

[50] Matsuo Y (2015). Fibronectin type III domain containing 5 expression in skeletal muscle in chronic heart failure—relevance of inflammatory cytokines. J. Cachexia Sarcopenia Muscle.

[51] Narayanan SA, Metzger CE, Bloomfield SA, Zawieja DC (2018). Inflammation-induced lymphatic architecture and bone turnover changes are ameliorated by irisin treatment in chronic inflammatory bowel disease. FASEB J..

[52] Metzger CE (2019). DSS-induced colitis produces inflammation-induced bone loss while irisin treatment mitigates the inflammatory state in both gut and bone. Sci. Rep..

[53] Shao L, Meng D, Yang F, Song H, Tang D (2017). Irisin-mediated protective effect on LPS-induced acute lung injury via suppressing inflammation and apoptosis of alveolar epithelial cells. Biochem. Biophys. Res. Com..

[54] Colaianni G (2017). Irisin prevents and restores bone loss and muscle atrophy in hindlimb suspended mice. Sci. Rep..

[55] Robling AG (2008). Mechanical stimulation of bone in vivo reduces osteocyte expression of Sost/sclerostin. J. Biol. Chem..

[56] Kobayashi K (2000). Tumor necrosis factor-α stimulates osteoclast differentiation by a mechanism independent of the ODF/RANKL–RANK interaction. J. Exp. Med..

[57] Lam J (2000). TNF-α induces osteoclastogenesis by direct stimulation of macrophages exposed to permissive levels of RANK ligand. J. Clin. Invest..

[58] Kulkarni RN, Bakker AD, Everts V, Klein-Nulend J (2012). Mechanical loading prevents the stimulating effect of IL-1β on osteocyte-modulated osteoclastogenesis. Biochem. Biophys. Res. Comm..

[59] Pathak JL (2015). Mechanical loading reduces inflammation-induced human osteocyte-to-osteoclast communication. Calf Tissue Int..

[60] Bokhari RS, Metzger CE, Allen MR, Bloomfield SA (2018). Daily acute bouts of weight-bearing during hindlimb unloading mitigate disuse-induced deficits in cancellous bone. Gravitational Space Res..

[61] Durbin SM (2014). Resveratrol supplementation preserves long bone mass, microstructure, and strength in hindlimb-suspended old male rats. J. Bone Min. Metab..

[62] Smith BJ (2005). Vitamin E provides protection for bone in mature hindlimb unloaded male rats. Calcif. Tissue Int..

[63] Cunningham HC (2018). Age-dependent bone loss and recovery during hindlimb unloading and subsequent reloading in rats. BMC Musculoskelet. Dis..

[64] Morey-Holton ER, Globus RK (2002). Hindlimb unloading rodent model: technical aspects. J. Appl. Physiol..

[65] Dempster DW (2013). Standard nomenclature, symbols, and units for bone histomorphometry: a 2012 update of the report of the ASMBR histomorphometry nomenclature committee. J. Bone Min. Res..

